# Supplementary material for: MEKK2 mediates aberrant ERK activation in neurofibromatosis type I
Source: Nat Commun. 2020 Nov 11;11:5704. doi: 10.1038/s41467-020-19555-6 (PMC7658220; doi:10.1038/s41467-020-19555-6)
Supplement: Supplementary file 1 — Supplementary Information [file 41467_2020_19555_MOESM1_ESM.pdf]

Supplementary information

MEKK2 mediates aberrant ERK activation in neurofibromatosis type I

Bok et al.

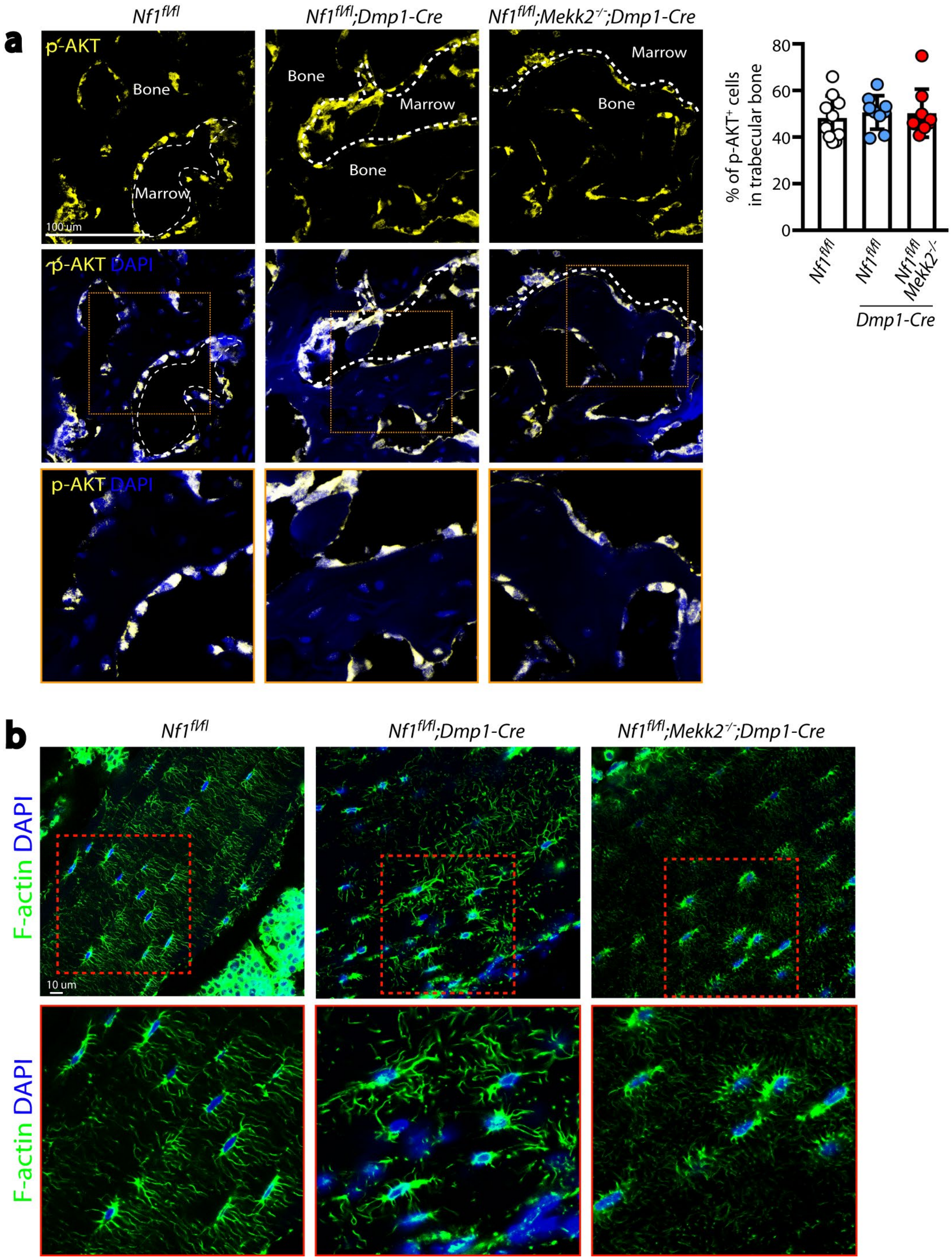

Supplementary Figure 1. **a** Representative images and quantification of immunostaining for p-AKT from 16 weeks old *Nf1$^{fl/fl}$*, *Nf1$^{fl/fl}$;Dmp1-Cre*, and *Nf1$^{fl/fl}$;Mekk2$^{-/-}$;Dmp1-Cre* mice. Scale bar indicates 100 μm. Three independent fields examined per mouse (*n* = 3 mice per group). mean ± s.d., one-way ANOVA with Tukey's multiple comparison test **b** Confocal images of

immunofluorescent F-actin staining in cortical bone of 16 weeks old *Nf1*$^{fl/fl}$, *Nf1*$^{fl/fl}$*;Dmp1-Cre*, and *Nf1*$^{fl/fl}$*;Mekk2*$^{-/-}$*;Dmp1-Cre* mice. Lower panels show enlarged views of the dotted red boxes. Scale bar indicates 10 µm. Nuclei are counterstained with DAPI (blue). Six independent fields examined per mouse (*n* = 3 mice per group). Source data are provided as a Source Data file.

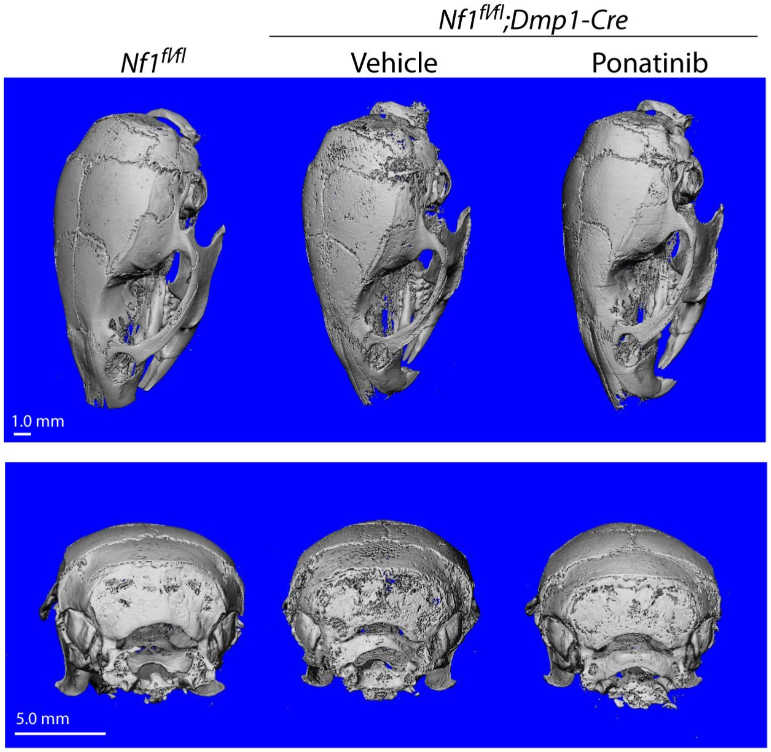

Supplementary Figure 2. Lateral view (upper) and posterior view (lower) of µCT scans of mouse skulls at 16 weeks old *Nf1*$^{fl/fl}$ and *Nf1*$^{fl/fl}$*;Dmp1-Cre* mice treated with vehicle or ponatinib.

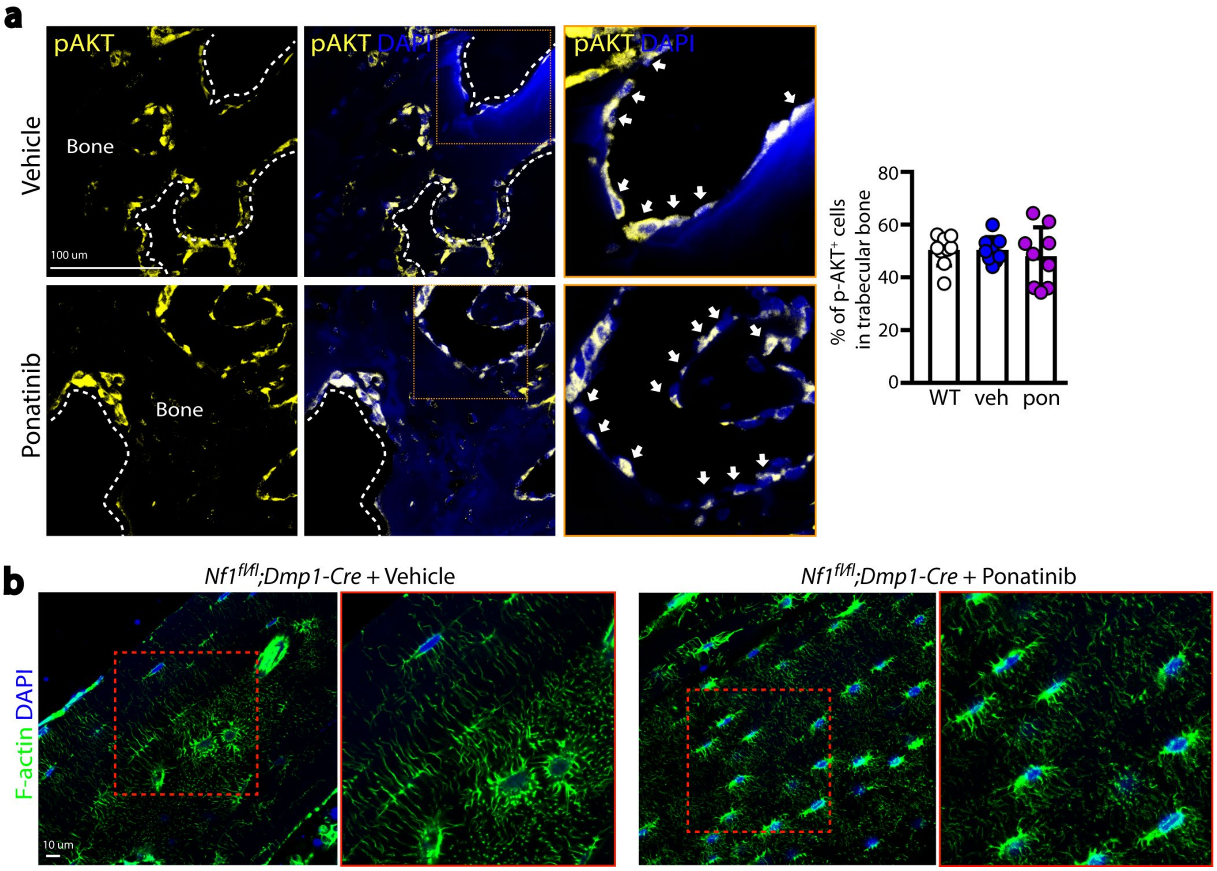

Supplementary Figure 3. a Representative images and quantification of immunostaining for p-AKT in femurs from 16 weeks old *Nf1$^{fl/fl}$;Dmp1-Cre* mice treated with vehicle or ponatinib. Scale bar indicates 100 μm. Three independent fields were examined per mouse (*n* = 3 mice per group). mean ± s.d., one-way ANOVA with Tukey's multiple comparison test **b** Confocal images of immunofluorescent F-actin staining in cortical bone from 16 weeks old *Nf1$^{fl/fl}$;Dmp1-Cre* mice treated with vehicle or ponatinib. Enlarged views of the dotted red boxes were displayed on the right from the original images. Scale bar indicates 10 μm. Nuclei are counterstained with DAPI (blue). Six independent fields examined per mouse (*n* = 3 mice per group). Source data are provided as a Source Data file.

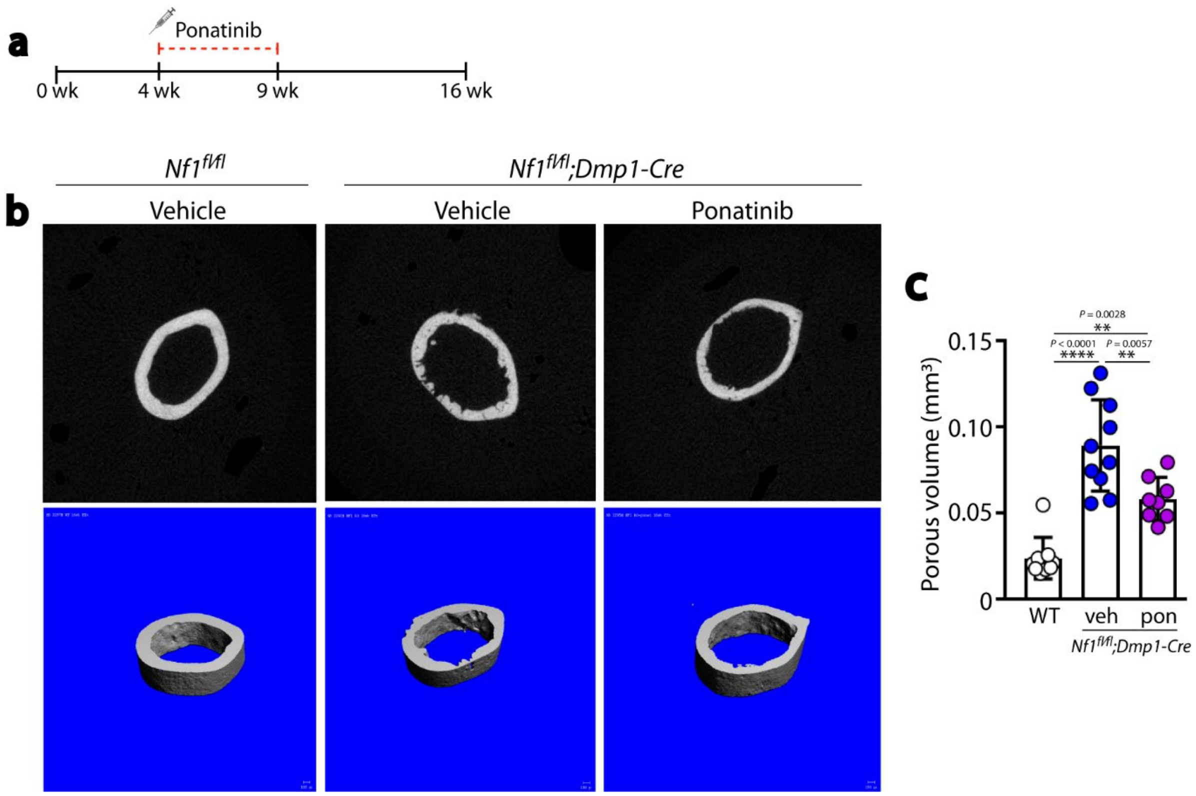

Supplementary Figure 4. a Experimental treatment scheme for durable effects. **b** Femurs from 16 weeks old *Nf1*$^{fl/fl}$ and *Nf1*$^{fl/fl}$*;Dmp1-Cre* mice treated with or without ponatinib were analyzed by μCT. Images of the midshaft cortical bone shown are representative of 8 or more mice per group. **c** Quantitative parameters are porous volume from WT (*Nf1*$^{fl/fl}$, *n* = 9), vehicle (veh, *n* = 10), and ponatinib-treated (pon, *n* = 8) group of *Nf1*$^{fl/fl}$*;Dmp1-Cre* mice. mean ± s.d., one-way ANOVA with Tukey's multiple comparison test. **P < 0.01; ****P < 0.0001. Source data are provided as a Source Data file.

Supplementary Figure 5

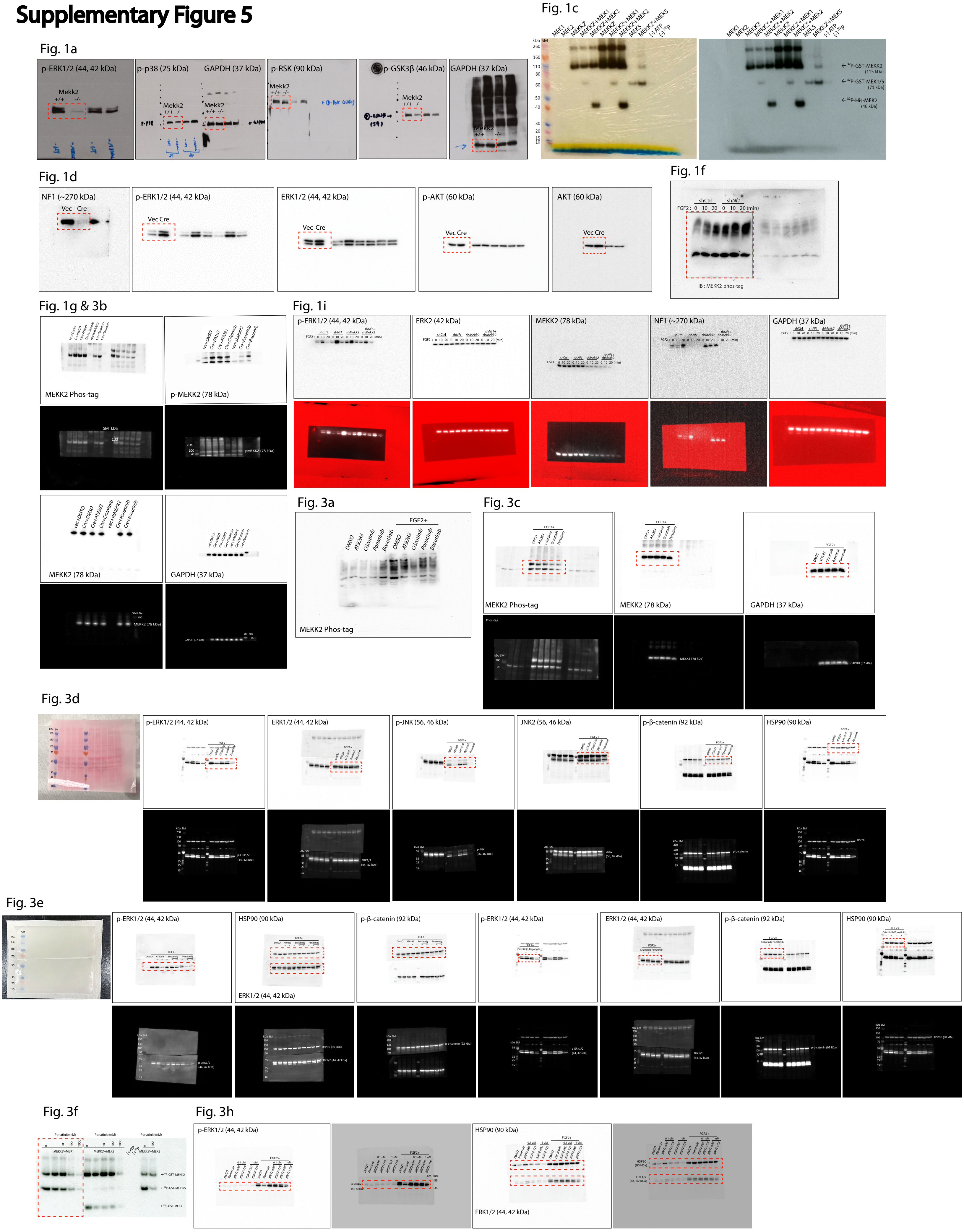

Supplementary Table 1. Primer sequences for qRT-PCR

Gene	Forward	Reverse
Mouse *β-actin*	GGC ACC ACA CCT TCT ACA ATG	GGG GTG TTG AAG GTC TCA AAC
Mouse *Osx*	ATG GCG TCC TCT CTG CTT GA	GAA GGG TGG GTA GTC ATT TG
Mouse *Runx2*	TAC AAA CCA TAC CCA GTC CCT GTT T	AGT GCT CTA ACC ACA GTC CAT GCA
Mouse *Bsp*	CAG GGA GGC AGT GAC TCT TC	AGT GTG GAA AGT GTG GCG TT
Mouse *Ocn*	GGG CAA TAA GGT AGT GAA CAG	GCA GCA CAG GTC CTA AAT AG
