# Supplementary material for: MEKK2 mediates aberrant ERK activation in neurofibromatosis type I
Source: Nat Commun. 2020 Nov 11;11:5704. doi: 10.1038/s41467-020-19555-6 (PMC7658220; doi:10.1038/s41467-020-19555-6)
Supplement: Supplementary file 3 — Reporting Summary [file 41467_2020_19555_MOESM3_ESM.pdf]

nature research

Corresponding author(s): Matthew Blake Greenblatt

Last updated by author(s): Sep 3, 2020

Reporting Summary

Nature Research wishes to improve the reproducibility of the work that we publish. This form provides structure for consistency and transparency in reporting. For further information on Nature Research policies, see Authors & Referees and the Editorial Policy Checklist .

Statistics

For all statistical analyses, confirm that the following items are present in the figure legend, table legend, main text, or Methods section.

n/a	Confirmed	
☐	☒	The exact sample size (*n*) for each experimental group/condition, given as a discrete number and unit of measurement
☐	☒	A statement on whether measurements were taken from distinct samples or whether the same sample was measured repeatedly
☐	☒	The statistical test(s) used AND whether they are one- or two-sided<br>*Only common tests should be described solely by name; describe more complex techniques in the Methods section.*
☒	☐	A description of all covariates tested
☐	☒	A description of any assumptions or corrections, such as tests of normality and adjustment for multiple comparisons
☐	☒	A full description of the statistical parameters including central tendency (e.g. means) or other basic estimates (e.g. regression coefficient) AND variation (e.g. standard deviation) or associated estimates of uncertainty (e.g. confidence intervals)
☐	☒	For null hypothesis testing, the test statistic (e.g. *F*, *t*, *r*) with confidence intervals, effect sizes, degrees of freedom and *P* value noted<br>*Give P values as exact values whenever suitable.*
☒	☐	For Bayesian analysis, information on the choice of priors and Markov chain Monte Carlo settings
☒	☐	For hierarchical and complex designs, identification of the appropriate level for tests and full reporting of outcomes
☒	☐	Estimates of effect sizes (e.g. Cohen's *d*, Pearson's *r*), indicating how they were calculated

Our web collection on statistics for biologists contains articles on many of the points above.

Software and code

Policy information about availability of computer code

Data collection	SCANCO Medical microCT 35 system (v4.05, Switzerland) was used for scanning and 3D analysis.<br>Carl Zeiss Zen 2.3 SP1 FP3 (black, v14.0.18.201, Germany) was used for immunofluorescence imaging analysis.<br>QuantStudio 6 Flex RT-PCR Software v1.3 was used for mRNA analysis.
Data analysis	GraphPad PRISM software (v7.04, La Jolla, CA) was used for statistical analysis.<br>SCANCO Medical microCT 35 system (v4.05, Switzerland) was used for scanning and 3D analysis.<br>Carl Zeiss Zen 2.3 SP1 FP3 (black, v14.0.18.201, Germany) was used for immunofluorescence imaging analysis.<br>QuantStudio 6 Flex RT-PCR Software v1.3 was used for mRNA analysis.

For manuscripts utilizing custom algorithms or software that are central to the research but not yet described in published literature, software must be made available to editors/reviewers. We strongly encourage code deposition in a community repository (e.g. GitHub). See the Nature Research guidelines for submitting code & software for further information.

Data

Policy information about availability of data

All manuscripts must include a data availability statement. This statement should provide the following information, where applicable:

- Accession codes, unique identifiers, or web links for publicly available datasets
- A list of figures that have associated raw data
- A description of any restrictions on data availability

All relevant data are available from the authors. Uncropped blots are provided in Supplementary Figure 5. Source data are provided with this paper. The source data underlying Figures 1b, 2b, 2d, 2f, 2g, 3g, 4b, 4d, 4f, 4g, Supplementary Figure 1a, 3a, and 4c are provided as a Source Data file.

Field-specific reporting

Please select the one below that is the best fit for your research. If you are not sure, read the appropriate sections before making your selection.

- [x] Life sciences
- [ ] Behavioural & social sciences
- [ ] Ecological, evolutionary & environmental sciences

For a reference copy of the document with all sections, see nature.com/documents/nr-reporting-summary-flat.pdf

Life sciences study design

All studies must disclose on these points even when the disclosure is negative.

Sample size

Generally, sample sizes were calculated on the assumption that a 30% difference in the parameters measured would be considered biologically significant with an estimate of sigma of 10-20% of the expected mean. Alpha and Beta were set to the standard values of .05 and 0.2, respectively.

Data exclusions

No exclusion

Replication

Yes, all experiments reported in the manuscript were replicated to confirm reproducibility.
1) Immunoblotting on mouse samples included at least 3 independent experiments, each with consistent results.
2) Animal studies were reproduced across at least 3 independent cohorts.
3) Analysis of serum included at least 8 independent samples and 5 independent samples per each genotype and experimental group, respectively.
4) Ponatinib in vivo treatment were reproduced across three independent experiments.
5) Immunostaining on mouse samples included at least 3 independent experiments and analyzed at least nine independent fields per each genotype and experimental group.

Randomization

For ponatinib treatment experiments, animals were randomized to treatment vs vehicle groups.
For other experiments, specimen were assigned to group based on genotype.

Blinding

Yes, µCT analysis, serum assay, and immunohistochemistry were performed by individuals (Alisha R. Yallowitz, Mark Eiseman, Michelle Cung, Ren Xu, Na Li) who were blinded to the nature of the mice under analysis (both what specific mouse strains or treatment groups were in the experiment and whether any individual mouse belonged to control versus experimental groups).

Reporting for specific materials, systems and methods

We require information from authors about some types of materials, experimental systems and methods used in many studies. Here, indicate whether each material, system or method listed is relevant to your study. If you are not sure if a list item applies to your research, read the appropriate section before selecting a response.

Materials & experimental systems

n/a	Involved in the study	
☐	☒	Antibodies
☐	☒	Eukaryotic cell lines
☒	☐	Palaeontology
☐	☒	Animals and other organisms
☒	☐	Human research participants
☒	☐	Clinical data

Methods

n/a	Involved in the study	
☒	☐	ChIP-seq
☒	☐	Flow cytometry
☒	☐	MRI-based neuroimaging

Antibodies

Antibodies used

Primary antibodies used for immunoblotting and immunofluorescence staining were specific for NF1 (cat. no. A300-140A, BETHYL, lot no. 3), MEKK2 (cat. no. A302-163A, BETHYL, lot no. 1), phospho-MEKK2 (Ser520) (cat. no. PA5-105898, Invitrogen), phospho-MEK1 (Thr286) (cat. no. 9127, Cell Signaling Technology), ERK1/2 (cat. no. 9102, Cell Signaling Technology, lot no. 20), phospho-ERK1/2 (Thr202/Tyr204) (cat. no. 4377, Cell Signaling Technology, lot no. 6), phospho-ERK1/2 (cat no. 4370, Cell Signaling Technology, lot no. 24), AKT (cat. no. 9272, Cell Signaling Technology, lot no. 27), phospho-AKT (Thr308) (cat. no. 13038, Cell Signaling Technology, lot no. 5), phospho-AKT (Ser473) (cat. no. 4060, Cell Signaling Technology, lot no. 25), phospho-p38 (Thr180/Tyr182) (cat. no. 9215, Cell Signaling Technology, lot no. 5), phospho-RSK (Ser380) (cat. no. 12032, Cell Signaling Technology, lot no. 1), phospho-GSK-3β (Ser9) (cat. no. 5558, Cell Signaling Technology, lot no. 3), phospho-β-catenin (Ser675) (cat. no. 4176, Cell Signaling Technology, lot no. 3), JNK2 (cat. no. 9258, , Cell Signaling Technology), phospho-JNK (Thr183/Tyr185) (cat. no. 4668, Cell Signaling Technology, lot no. 11), HSP90 (cat. no. sc-515081, Santa Cruz Biotechnology, lot no. H1815) and GAPDH (cat. no. sc-25778, Santa Cruz Biotechnology, lot no. I3015).
The phospho-MEKK2 polyclonal antibody was generated by immunizing rabbit with phospho-MEKK2 peptide.

	Primary antibodies were detected with goat anti-mouse IgG HRP (cat no. 31430, Invitrogen), goat anti-rabbit IgG HRP (cat no. 31460, Invitrogen), and goat anti-rabbit IgG Alexa fluor 633 (cat no. A-21070, Invitrogen, lot no. 1889306).
Validation	The following validation method was conducted: for IHC, secondary antibody was added alone without primary mouse antibody addition as negative control. The manufacturer has validated these antibodies for use in the same species and assay format.

Eukaryotic cell lines

Policy information about cell lines

Cell line source(s)	Validation for drug efficacy was initially done with primary human mesenchymal stromal cells from Lonza (cat no. PT-2501) and Saos-2 cells, a commercially available human osteosarcoma cell line, from ATCC (cat no. ATCC® HTB-85™).
Authentication	These cells were used directly from the manufacturer (Lonza and ATCC) who performed authentication.<br>These cell lines were not authenticated by the authors.
Mycoplasma contamination	Cells were tested for mycoplasma using the PlasmoTest™ - Mycoplasma Detection Kit from InvivoGen (cat no. rep-pt1) and PCR analysis. All cells were confirmed to be free of mycoplasma.
Commonly misidentified lines<br>(See ICLAC register)	No commonly misidentified cell lines were used.

Animals and other organisms

Policy information about studies involving animals; ARRIVE guidelines recommended for reporting animal research

Laboratory animals	Floxed Nf1 (Nf1fl/fl) mice (Stock 017639) and dentin matrix protein 1-Cre (Dmp1-Cre, Stock 023047) were purchased from Jackson Laboratories. Mekk2-/- mouse strain were a originally generated by Bing Su (Shanghai JiaoTong University School of Medicine, Shanghai, China). Transgenic mice expressing Cre recombinase under control of the Dmp1 promoter (Dmp1-Cre) were bred with Nf1fl/fl and/or Mekk2-/- mice. All mice used were backcrossed more than six generations onto the C57BL/6 background. For in vivo experiment, all mice were 16 weeks old female and male mice. Primary osteoblasts were extracted from 5 days old female and male pups.<br>All mice were maintained on a C57Bl6/J background throughout the study. All animals were maintained a 12 hr light/dark cycle, temperatures of 64-79°F (~18-26°C) with 40-60% humidity in accordance with the NIH Guide for the Care and Use of Laboratory Animals and were handled according to protocols approved by the Weill Cornell Medical College subcommittee on animal care (IACUC).
Wild animals	This study did not involve wild animals.
Field-collected samples	This study did not involve field-collected samples.
Ethics oversight	Animals were maintained in accordance with the NIH Guide for the Care and Use of Laboratory Animals and were handled according to protocols approved by the Weill Cornell Medical College subcommittee on animal care (IACUC). Animals were ethically euthanized by CO2 for primary cell isolation and analyses or morphological studies.

Note that full information on the approval of the study protocol must also be provided in the manuscript.
